# Exploring Factors Associated with Chinese-Americans’ Willingness to Receive an Additional Hypothetical Annual Dose of the COVID-19 Vaccine

**DOI:** 10.3390/vaccines11010185

**Published:** 2023-01-15

**Authors:** Ming Li, Bo Kyum Yang, Zuojin Yu, Lin Zhu, Xuewei Chen, Gary L. Kreps, Radhika Kansangra

**Affiliations:** 1 Department of Health Sciences, Towson University, Towson, MD 21252, USA; 2 Center for Asian Health, Lewis Katz School of Medicine, Temple University, Philadelphia, PA 19122, USA; 3 School of Community Health Sciences, Counseling and Counseling Psychology, Oklahoma State University, Stillwater, OK 74077, USA; 4 Center for Health & Risk Communication, George Mason University, Fairfax, VA 22030, USA

**Keywords:** Chinese-Americans, annual COVID-19 vaccine, intention

## Abstract

Chinese-Americans are one of the largest groups of Asian-Americans in the US with distinctive behavioral and cultural characteristics that influence health service use. Although Chinese-Americans have significantly higher COVID-19-related mortality rates, relative to other racial and ethnic groups, limited literature is available examining their willingness to accept the COVID-19 vaccine. With recent development of the combination influenza-COVID-19 vaccine by biotechnology companies to mitigate COVID-19 infection, we examined factors associated with Chinese-Americans’ acceptance of hypothetical annual doses of COVID-19 vaccination before the vaccine rollout. A total of 241 Chinese-Americans who received at least one dose of the COVID-19 vaccine completed an online questionnaire developed and based on health behavior theories. Our results indicated that Chinese-American participants who were satisfied with their prior COVID-19 vaccination experience, who had more accurate knowledge and perceived higher susceptibility of getting COVID-19, were more willing to receive the annual COVID-19 vaccine in the future. The findings of our current study may be used to guide the development of strategic messages to promote uptake of the annual COVID-19 vaccine by Chinese-Americans in the U.S.

## 1. Introduction

The COVID-19 vaccine is a safe and effective way to build immunity against COVID-19 infection and variants [1]. Up to date, 84.1% Americans aged five or above have received at least one dose of the COVID-19 vaccine, and 72% have completed a primary series of the vaccine [2]. The virus that causes COVID-19 has evolved over time. Up until August 2022, the Omicron subvariants BA.4, BA.4.6, and BA.5 were dominant in the U.S. [3]. To protect people from getting seriously ill and even dying due to COVID-19 and its evolving variants, it is important for all eligible individuals to stay up to date with the recommended number of boosters. The Centers for Disease Control and Prevention (CDC) recommends that everyone aged five years and older should get one vaccine booster after completing their COVID-19 vaccine primary series. In addition, adults aged 50 years and older or with certain health conditions should get a second booster 4 months after the first booster [4].

“Booster shots” is a term used as by health authorities (e.g., the U.S. Food and Drug Administration and CDC officials), which suggests that the vaccine may need to be regularly repeated to maintain protection against the infection [5]. The emergence of new SARS-CoV-2 variants and decreases in immunity are very likely to continue to require regular boosters or annual shots, like the influenza vaccines, which are modified annually to target the circulating influenza strains [5]. For example, scientists are working on the development of a combined mRNA vaccine against COVID-19 and influenza [6]. Biotechnology companies, such as Moderna and Novavax, have entered clinical development of a combination influenza-COVID-19 vaccine, which makes an annual booster against COVID-19 possible [5,7,8,9,10]. Future winter surges of COVID-19 have been predicted by five years of epidemiological models which suggest that COVID-19 is likely becoming another endemic respiratory virus that may require annual or intermittent booster immunization for the management and control of the pandemic due to the global mutations and the success of the authorized vaccine [11,12]. While the scientific evidence for annual vaccination is promising, this strategy needs population acceptance and public health vaccination promotion efforts [13]. It is critical to understand an individual’s intentions concerning potential annual COVID-19 vaccination and the factors associated with their vaccination decisions. Although numerous studies have assessed the factors affecting people’s willingness or hesitancy to receive the COVID-19 vaccine, the majority of these studies have focused only on the primary vaccination series and boosters [14]. Intentions toward hypothetical annual COVID-19 vaccination is largely unknown. Among the limited literature, racial/ethnic minorities were found to be more likely to have a lower vaccination willingness than White respondents when it comes to COVID-19 annual vaccine in both the U.S. and Canada [9,13]. 

Chinese-Americans make up the largest subgroup of Asian-Americans who often have limited English language proficiency and significant cultural barriers [15]. Up until July 2022, there were 2,225,573 Asian-Americans infected with COVID-19 and 24,089 deaths within this population due to COVID-19 [16]. Among these, Chinese-Americans particularly experienced significantly higher mortality rates attributed to COVID-19 than non-Hispanic Whites after adjusting for age, other demographic characteristics, and comorbidities [17,18]. This high mortality may be due to a limited access to linguistically and culturally appropriate health care, delay in or avoidance of seeking care due to worry about immigration status, or reluctance to or fear of leaving home for care or testing due to increasing xenophobia and harassment toward Chinese-Americans [17,19]. During the COVID-19 pandemic, Chinese-Americans experienced increasing anti-Asian hate in the form of racism and discrimination, and were blamed for spreading COVID-19 infections [20]. Fear of safety at vaccination sites and on public transportation, confusion about vaccination eligibility, concerns about health and economic impacts of vaccine side effects, medical mistrust, low health literacy about the disease and the benefits of vaccination have made Chinese communities particularly vulnerable to the spread of COVID-19 and could potentially create barriers to receiving the COVID-19 vaccine [19,21]. 

To the best of our knowledge, however, evidence concerning Chinese-Americans’ perspectives toward COVID-19 vaccine is limited. To address this gap in the literature, the purpose of the present study is to (1) assess intention to receive an additional hypothetical annual does of COVID-19 vaccination among Chinese-Americans who have received at least one dose of the COVID-19 vaccine; and (2) identify factors associated with their annual vaccination intention. 

## 2. Materials and Methods

### 2.1. Participants and Recruitment

This study was approved by the Towson University Institutional Review Board. Participation criteria included (1) Chinese-Americans who were living in the U.S. when filling out the survey, (2) were 18 years of age or older, and (3) had received at least one shot of COVID-19 vaccine. Qualtrics was used as the platform to collect survey data. Participants accessed the survey via a link or QR code from 5 June 2021 to 13 June 2021, when Delta variant became the dominant variant in the U.S. and a third wave of infections began. Responses were anonymous and participation was voluntary. Participant consent was obtained via information sheets. To de-identify participants when collecting their names and emails for respondent incentive (a $10 electronic gift card) distribution, all participants were provided with a link to a separate survey to enter their name and email. We used convenience sampling and promoted our study through social media, fliers posted in Asian stores and restaurants, and by networking with Asian-American churches and community organizations. A total of 374 participants filled out the survey. After excluding incomplete surveys, non-Chinese-American participants, and those who did not receive any shot of COVID-19 vaccine, a total of 241 respondents was included in the analytic models. We conducted a power analysis to calculate the estimated sample size. Our calculation indicates a sample size of 245 is needed for estimating the binary outcome variable with 95% confidence interval, an estimated 80% of respondents who would be willing to receive annual vaccination, and a 5% margin of error [22]. Our final sample size included in the analysis was 241, which was very close to 245. 

### 2.2. Survey Development

We developed a 20-min online survey adapted from the questionnaires published in the literature [23,24,25,26,27] based on health behavior theories and the Expectation Confirmation Model (ECM), a model that has been widely used in the marketing domain and shows how customers’ repurchase intentions depend on their level of satisfaction with the purchased product [28]. The intention to seek annual vaccination is similar to repurchase intention [29]. Satisfaction level with previous COVID-19 vaccination may influence decision making about receiving annual boosters [30]. We included demographic factors (e.g., age, gender, educational level, and socio-economic position) [31,32], and cognitive constructs from health behavior theories, such as the Health Belief Model (HBM) and the Theory of Planned Behavior (TPB), as potential factors in this study because they have been widely used in the context of vaccination intention, such as influenza and COVID-19 vaccine [23,24,33,34,35,36]. We adopted all constructs from the HBM. Previous literature also showed that subjective norms and self-efficacy fromTPB were significantly associated with COVID-19 vaccine intention [36]. 

Besides cognitive constructs adapted from theories, appropriate knowledge is the primary foundation of an individual’s behavior. Previous studies documented that knowledge plays an important role in protection behaviors against the pandemic, such as wearing facemasks, using hand sanitizer, and receiving the COVID-19 vaccine [37,38]. Specifically, COVID-19 vaccination knowledge has been found associated with attitude and acceptance of vaccination in diverse populations [38,39,40,41,42]. Thus, our survey also measured Chinese-Americans’ COVID-19 vaccination knowledge. 

To ensure the face validity, the survey was reviewed by experts on health behavior, healthcare management, and health disparities. The survey was then revised based upon their comments. The survey was originally developed in English and then translated into Chinese for the participants who preferred to use the Chinese language to fill out the survey. Translation and back translation procedures were utilized to ensure face and conceptual equivalence and cultural relevance [43]. Specifically, the English survey was translated into Chinese independently by two bilingual, native Mandarin speakers (M.L. and Z.Y.). After translations, two translators met and reviewed these two versions to resolve any discrepancies. After that, the research team invited a Chinese-American community member who is also a bilingual speaker and who back translated the Chinese version into English, being blind to the original English survey. This back translation survey then was rated for equivalence to the original English version by research team. 

### 2.3. Measures

Intention to receive boosters was measured by a single item (“If a seasonal COVID-19 vaccine every year is available to provide the best protection against COVID-19, how likely would you be to take it again?”), with responses provided on a 5-point scale (1 = very unlikely to 5 = very likely). 

Satisfaction with previous shots of COVID-19 vaccination was measured by one item, “To what extent are you satisfied with your experience of getting the COVID-19 vaccine? 1 = not at all satisfied; 5 = extremely satisfied”.

Perceived susceptibility of getting COVID-19 was measured with 4 items (e.g., “I am at risk of getting COVID-19. 1 = Strongly disagree; 5 = Strongly agree”) adapted from previous literature [24,25]. The items used to examine perceived susceptibility showed a moderate reliability (Cronbach’s α = 0.724) and adequate validity (RMSEA = 0, CFI = 1.000, SRMR = 0.002).

Perceived severity of getting COVID-19 was measured with 6 items (e.g., I believe that COVID-19 has serious negative consequences. 1 = Strongly disagree; 5 = Strongly agree) adapted from previous literature [24,25]. The items indicated a good reliability (Cronbach’s α was 0.902) and adequate validity (RMSEA = 0.035, CFI = 0.998, SRMR = 0.019).

Perceived benefits of getting the COVID-19 vaccine were measured with 6 items adapted from previous literature (e.g., If I get the vaccine, it will help to protect my family from getting COVID-19. 1 = Strongly disagree; 5 = Strongly agree) [23,24]. The items showed a good reliability (Cronbach’s α was 0.884) and adequate validity (RMSEA = 0, CFI = 1, SRMR = 0.012).

Perceived barriers to getting the COVID-19 vaccine were measured with 4 items adapted from previous literature (I have concerns about possible side effects of COVID-19 vaccine. 1 = Strongly disagree; 5 = Strongly agree) [23,24]. The items showed a good reliability (Cronbach’s α = 0.855) and adequate validity (RMSEA = 0.063, CFI = 0.996, SRMR = 0.018).

Self-efficacy for getting the COVID-19 vaccine was measured with 3 items adapted from previous studies (e.g., “I feel confident in making an appointment to receive a COVID-19 vaccine.” 1 = Strongly disagree; 5 = Strongly agree) [24]. Cronbach’s α was 0.824. Confirmatory factor analysis (CFA) results showed a saturated model due to the three items for this construct, and all three items were significantly related to the construct (*p* < 0.001).

Subjective norms for getting the COVID-19 vaccine refers to perceived social pressure from important person or group of people for an individual’s decision on vaccination. This construct was measured with 3 items adapted from previous studies (e.g., “Most people who are like me will get vaccinated for COVID-19.” 1 = Strongly disagree; 5 = Strongly agree) [24]. Cronbach’s α was 0.846. CFA result showed a saturated model due to the three items for this construct, and all three items were significantly related to the construct (*p* < 0.001).

Ten items adapted from previous literature were used to measure cue to actions (e.g., “tested positive for COVID-19” “family or close friend tested positive for COVID-19,” and “someone you knew passed away due to COVID-19”). If participants opted for any of these choices, they were coded as “yes” for cue to actions of COVID-19 vaccine. 

Knowledge of COVID-19 vaccine was measured by 7 false/true questions developed by the research team based on educational materials from the CDC website [26] (e.g., “after getting a COVID-19 vaccine, I will test positive for COVID-19 on a viral test (correct answer: false”). A knowledge score was calculated by summing participants’ correct answers. 

Failure to receive the preferred type of COVID-19 vaccine in previous shot(s) was measured using two questions: “1. What is the manufacturer/developer of the COVID-19 vaccine you actually received or will potentially receive? (Pfizer-BioNTech/Moderna/Johnson and Johnson’s Janssen/Novavax/AstraZeneca/Other, please specify/I don’t know)” and “2. What is your preferred manufacturer/developer of the COVID-19 vaccine? (Pfizer-BioNTech/Moderna/Johnson and Johnson’s Janssen/Novavax/AstraZeneca/Other, please specify/No preference)”. Participants were categorized as “failure to receive the preferred type of COVID-19 vaccine in previous shot(s)” if their responses for the first question were not consistent with those for the second one. For the participants who picked the same types of vaccine for both questions or chose “no preference” for the second question, they were not considered as “failure to receive the preferred type of COVID-19 vaccine”.

Sociodemographic information (i.e., age, gender, birthplace, marital status, educational level, religion, employment, and household income) was also collected in the survey because it may also influence Chinese-Americans’ decision to receive the booster vaccines.

### 2.4. Data Analysis 

The frequency, percentages, means, and standard deviations of variables were examined with descriptive statistics. Psychometric testing was conducted for cognitive constructs with multiple items. Specifically, Cronbach’s alpha was used to check construct reliability, and confirmatory factor analysis (CFA) was used to check the unidimensional validity of each construct with 3 items or more based on three fit indices: the root mean square error of approximation (RMSEA), comparative fit index (CFI), and standardized root mean residual (SRMR). An RMSEA < 0.08, a CFI > 0.90, and an SRMR < 0.06, were adopted as the cut-off point for an adequate model fit [44]. CFA was not applied to all scales because some scales had less than 3 items.

Bivariate analyses were conducted to examine the relationship between outcome variables (i.e., intention to receive annual vaccine) and predictors. Specifically, we used modified Poisson regressions to examine the associations. Modified Poisson regression is considered as a better approach compared to logistic regression when using common binary outcomes with more than 10% of occurrence [45,46]. Demographic variables with significant bivariate test results (*p* < 0.05) were included in a multivariable modified Poisson regression model to assess factors associated with Chinese-Americans’ willingness to receive an annual COVID-19 vaccine. Constructs adopted from theories were all included in the model no matter whether significant or unsignificant results in bivariate analyses. The choice of survey language was also included in the final model because it is considered as an important factor which may influence immigrants’ decision making in healthcare. STATA 17 was used to conduct all analyses with the alpha level of *p* < 0.05. 

## 3. Results

### 3.1. Demographic Characteristics

Table 1 presents the characteristics of the 241 Chinese-Americans who participated in this study. In our sample, 159 of the respondents were female (66.0%), and 97 were male (32.8%). Two participants refused to answer this question (0.8%), and one reported other as gender (0.4%). The average age of our respondents is 42.7 years old. The majority of the participants (*n* = 220, 93.2%) were born outside of the U.S. More than half of the participants had a graduate degree (*n* = 152, 63.1%). More than half of our participants were employed (*n* = 146, 60.6%) and had an annual household income above $75,000 (*n* = 129, 54.9%). Less than half of the participants (*n* = 105, 44.1%) affiliated with a particular religion (e.g., Catholic, Protestant, Jewish, Muslim, Hindu, Buddhist, and other). Most participants (*n* = 138, 57.3%) chose English as the language to fill out the survey, while the rest of the participants (*n* = 103, 42.7%) chose the Chinese version of the survey.

The majority of the participants (86.3%) reported that they were willing to receive the boosters, while the rest of the participants (13.7%) were not willing to receive annual boosters even if they had previously received shots of the COVID-19 vaccine. The mean of satisfaction is 4.1 with a range from 1 to 5. Most participants (83.0%) felt either very satisfied or extremely satisfied with their experience with the COVID-19 vaccination. However, more than fifty-one participants (16.2%) reported that they did not receive their preferred type of COVID-19 vaccine. 

### 3.2. Chinese-Americans’ Intention to Receive Annual COVID-19 Vaccine

As shown in Table 2, participants with higher satisfaction for the previous COVID-19 vaccination (adjusted PR = 1.104, CI: 1.023, 1.191), perceived higher susceptibility of getting COVID-19 (adjusted PR = 1.082, CI: 1.001, 1.169), and having more accurate knowledge of COVID-19 vaccine (adjusted PR = 1.046, CI: 1.001, 1.094) were more willing to receive an annual COVID-19 vaccine. Table 3 presents the correct rate for each knowledge item. 

## 4. Discussion

The current survey study examined Chinese-Americans’ intention to receive an additional hypothetical annual COVID-19 vaccine dose and explored the factors associated with this intention. The findings of our study indicated that while most Chinese-American participants were willing to accept an annual COVID-19 vaccine, nearly 14% of the participants were hesitant to receive an annual vaccine. The factors significantly associated with acceptance of the hypothetical annual COVID-19 vaccine included satisfaction with prior COVID-19 vaccination experience, knowledge regarding COVID-19 vaccine, and perceived risk of getting COVID-19. 

The majority of our participants (86.3%) were willing to get vaccinated annually against COVID-19. Our findings are similar with a cross-sectional survey study conducted in Europe, which indicated that 82.4% of participants from a German-speaking region were willing to receive annual vaccinations. However, the percentage from our study is higher than that of a survey study to examine Canadian adults’ willingness to accept annual vaccines, which indicated that 64.7% of the respondents reported the acceptance of an annual COVID-19 vaccine dose [13]. The high acceptance rate among Chinese-American participants in our study may suggest that the idea of making COVID-19 vaccination an annual routine is feasible and acceptable. However, we cannot ignore that nearly 14% of the Chinese-American participants were hesitant to receive an annual vaccine even though they had received at least one dose previously. Our study also reinforces that even though people received the initial primary series of the vaccine, this does not mean that they plan to receive continuous doses without any concerns. If annual COVID-19 vaccination is required, it is important to consider the individual’s willingness and understand their potential concerns regarding the vaccination. In addition, a clear guideline from health authorities, advocates by governments, and recommendations from healthcare providers will play important roles in improving vaccine acceptance rates.

Notably, our results indicated that Chinese-American participants who were satisfied with prior COVID-19 vaccination were more likely to accept the annual vaccine than those who were not satisfied. If the annual COVID-19 vaccine is recommended in the future, it will be important to ensure that those who plan to receive it act on their intentions and efforts must be taken to encourage those who are hesitant to get it to change their minds. It is critically important to provide Chinese-Americans with high-quality and accessible vaccine services, including providing easy scheduling of vaccination appointments through patient/community navigation, convenient access to vaccination sites (such as mobile or community-based vaccination programs), translation services, culturally and linguistically appropriate vaccine educational materials and services, good vaccination management, and efficient pre-vaccination communication with pharmacists, nurses, or doctors [47]. 

Along with satisfaction, a high level of knowledge of the COVID-19 vaccine was positively and significantly associated with participants’ intention to accept an annual vaccine. This finding is consistent with previous studies exploring other population members’ intentions to receive the COVID-19 vaccine. A nationally representative survey of 1027 adults in the U.S. found that many respondents, especially racial/ethnic minorities and people with lower education levels, overestimated the side effects of the COVID-19 vaccine, significantly underestimated the size/scale of the clinical trials for the COVID-19 vaccine, and perceived that the vaccine contained live coronavirus [48]. Our results indicated similar misconceptions. For example, only slightly higher than half of the Chinese-American participants (64.3%) answered correctly on the survey knowledge item about COVID-19 test results after vaccination; only 63.5% of the participants answered correctly on the item about fertility after receiving the COVID-19 vaccine. Our findings suggest that among the people who already received at least one dose, dangerous myths concerning the COVID-19 vaccine still exist. 

Compared to other populations outside of the U.S., our sample perceived low-level knowledge of COVID-19 and vaccines. For example, a survey study conducted among elderly people in Southern Italy indicated a satisfactory level of knowledge about COVID-19 and related control measures (with a correct answer rate ≥ 81.5%) [37]. Another study conducted on mainland Chinese university students also showed a higher self-rated knowledge level (mean score: 5.62, ranging from 1–7) toward COVID-19 vaccination [39].

Thus, delivering accurate vaccine information to Chinese-Americans is critically important to counter the myths and encourage vaccination. Educational efforts are needed to help racial/ethnic minorities (including Chinese-Americans) to better understand the benefits of COVID-19 vaccination and to eliminate misconceptions about the vaccine. Informing members of this population about the safety of vaccination and addressing their concerns about the vaccine may decrease hesitancy about their receiving of continuous annual vaccine doses. For example, future public health outreaches for Chinese-American communities are needed to educate the uninformed regarding the potential benefits and immunization process of annual doses of the COVID-19 vaccine. 

Our results also showed that Chinese-American participants’ intention to accept an annual COVID-19 vaccination was significantly associated with greater perceived susceptibility. This finding was consistent with previous literature regarding COVID-19 vaccine hesitancy and annual influenza vaccinations [40,49]. The number of people who have experienced COVID-19 infection continues to grow. More and more people are infected more than once. If annual vaccination is promoted in the future, it is important to deliver targeted messages to people who perceived different levels of risk from the virus. According to Rothman and colleagues’ use of gain- and loss-frame messages to promote healthy behavior, gain-framed appeals may be more effective when individuals perceive high risk for a disease, whereas loss-framed appeals are more effective when individuals perceive low disease risk [49,50]. Future public health campaigns may utilize this strategy if annual COVID-19 vaccination is widely approved for mass vaccination against COVID-19 variants in the future. 

The previous literature indicates that English proficiency is an important factor associated with Asian-Americans’ perceptions of COVID-19 vaccination [51]. The Asian-American participants with limited English proficiency may face more challenges in obtaining accurate and timely information about the pandemic as well as updates of vaccines [51,52]. In this study, however, selecting a preferrable language for the survey was not a significant factor associated with participants’ willingness to receive an annual COVID-19 vaccine. This may be because the majority of our participants received high-level education (with 61.5% of the participants completing graduate school or above) and they were proficient in English (with 56.3% of the respondents choosing English to answer the survey). Thus, for those who were proficient in both English and their native language, the use of language may be a personal preference rather than being associated with healthcare decision making. To examine the association between language proficiency and vaccination hesitancy, future studies may need to recruit more participants with limited English proficiency and use a survey scale to measure English proficiency. 

This study had some limitations. First, our sample was not recruited using random selection methods. More than half of our participants had received a high level of education and had a high income. However, these sample factors reinforce that vaccine hesitancy issues are not just limited to those with lower education and income levels. The findings of this study may not be generalized to all Chinese-Americans living in the U.S. Our relatively small sample size tempers our ability to generalize our findings to the entirety of the U.S. or other countries. Second, this study did not explore the reasons behind the unwillingness or willingness to receive the annual COVID-19 vaccine. Future research should identify specific motivators and barriers to continuing vaccination. In addition, as the annual COVID-19 vaccine is a hypothetical situation, participants’ responses were based on a hypothetical scenario. Their opinions may change if and when the actual situation may occur, where epidemiological data and vaccination policy may play an important role in their decision making. Fourth, this study did not assess the association between annual vaccination intention and other cognitive constructs from TBP (e.g., attitudes and perceived behavioral control). Future research is needed to test the application of the full theory. Fifth, although construct items were adopted from a previously validated instrument, the questionnaire was not piloted before full implementation. However, the psychometric testing showed that our instrument is valid and reliable. Fifth, due to the nature of cross-sectional design, the causal relationship of the effect cannot be concluded [53]. Future research might build upon this study by utilizing repeated survey administrations to track trends over time in annual vaccine hesitancy among Chinese-Americans. Lastly, it is important to note that, the question item measuring our dependent variable did not specify whether it meant taking up vaccines in an ad hoc manner (e.g., single booster) or annually. Therefore, there is a possibility that some participants may have misinterpreted the meaning of annual vaccine, confusing this variable with boosters, especially in the data collection period when the boosters were not available. Despite the above limitations, this study provides important insights into the factors associated with Chinese-Americans’ intention to receive an annual COVID-19 vaccine. To the best of our knowledge, this is a first-of-its-kind study examining Chinese-Americans’ willingness to accept a hypothetical annual COVID-19 vaccine. The findings of the present study indicated that most Chinese-American participants were willing to accept the annual COVID-19 vaccine. Those who were satisfied with their prior COVID-19 vaccination experience, who had more accurate knowledge and perceived higher susceptibility of getting COVID-19, were willing to receive the annual COVID-19 vaccine in the future. The findings of our current study may be used to provide recommendations when developing strategic messages to promote annual COVID-19 vaccination, specifically targeting Chinese-Americans. 

## 5. Conclusions

To the best of our knowledge, this is a first-of-its-kind study in the assessment of Chinese-Americans’ willingness to accept a hypothetical annual COVID-19 vaccine. Our findings indicated that most Chinese-American participants were willing to get an annual COVID-19 vaccine. In addition, our findings highlight the importance of enhancing the positive experience of the previous vaccination, accurate knowledge regarding COVID-19 vaccination, and perceived risk of COVID-19 infections among Chinese-Americans to promote annual COVID-19 vaccination. If annual COVID-19 vaccination is required in the future, a clear guideline from health authorities, advocation by governments, public health educational efforts, and recommendations from healthcare providers will play important roles in improving vaccine acceptance rates among Chinese-Americans.

## Figures and Tables

**Table 1:** Socio-demographic characteristics and survey responses of study participants who are willing to receive the annual dose of COVID-19 vaccine compared to those who are unwilling to receive the vaccine (*n* = 241).

Variable	Willingness to Accept the Vaccine (*n* = 208)	Unwillingness to Accept the Vaccine (*n* = 33)	Prevalence Ratio (PR)	*p*-Value
Age	42.7 (16.1) ^a^	44.1 (16.0)	0.999	0.581
Gender				
Female	137 (66.8%) ^b^	22 (66.7%)	Ref	
Male	68 (33.2%)	11 (33.3%)	1.001	0.985
Born in the U.S.				
No	189 (92.7%)	31 (96.9%)	Ref	
Yes	15 (7.4%)	1 (3.1%)	1.091	0.214
Education				
High school graduate or below	18 (8.7%)	2 (6.1%)	Ref	
Some college or associate degree	31 (14.9%)	2 (6.1%)	1.044	0.622
Four-year college degree	31 (14.9%)	5 (15.2%)	0.957	0.660
Graduate school or above	128 (61.5%)	24 (72.7%)	0.936	0.421
Marital status				
Married/living with a partner	165 (80.5%)	27 (81.8%)	Ref	
Other	40 (19.5%)	6 (18.2%)	1.012	0.854
Employment				
Employed	124 (59.6%)	22 (66.7%)	Ref	
Other	84 (40.4%)	11 (33.3%)	0.961	0.430
Religion				
Affiliated	91 (44.0%)	14 (45.2%)	Ref	
Unaffiliated/none	116 (56.0%)	17 (54.8%)	0.994	0.901
Annual household income				
$0 to $19,999	43 (21.2%)	6 (18.8%)	Ref	
$20,000 to $74,999	49 (24.1%)	8 (25.0%)	0.980	0.786
$75,000 or more	111 (54.7%)	18 (56.3%)	0.981	0.759
Use of the survey language				
English	117 (56.3%)	21 (63.6%)	Ref	
Chinese	91 (43.8%)	12 (36.3%)	1.042	0.418
Failure to receive the preferred type of COVID-19 vaccine in previous shot(s)				
Yes	35 (16.8%)	4 (12.1%)	Ref	
No	173 (83.2%)	29 (87.9%)	1.048	0.447
Satisfaction with previous shots of COVID-19 vaccine	4.1 (1.0–5.0) ^c^	3.6 (1.0–4.0)	1.154	<0.001
Perceived benefits of receiving COVID-19 vaccine	4.3 (2.7–5.0)	4.0 (2.8–5.0)	1.242	0.001
Perceived barriers of receiving COVID-19 vaccine	3.2 (1.0–5.0)	3.4 (1.8–5.0)	0.957	0.151
Perceived susceptibility of getting COVID-19	3.3 (1.0–5.0)	2.9 (2.0–4.0)	1.107	0.003
Perceived severity of getting COVID-19	4.1 (1.0–5.0)	3.6 (2.3–5.0)	1.109	0.015
Self-efficacy in receiving COVID-19 vaccine	4.3 (2.3–5.0)	4.1 (2.7–5.0)	1.063	0.155
Subjective norms	4.2 (2.3–5.0)	3.9 (2.7–5.0)	1.166	0.003
Knowledge of COVID-19 vaccine	5.5 (0–7.0)	4.2 (0–7.0)	1.068	0.003
Cue to action				
No	116 (55.7%)	23 (69.7%)	Ref	
Yes	92 (44.2%)	10 (30.3%)	1.081	0.120

Note. ^a^ mean age (SD); ^b^ *n* (%); ^c^ mean score (range); Ref = reference; *p*-value represents a significance level (<0.05) on the bivariate association between each predictor and willingness to accept the annual dose of COVID-19 vaccine; sum of the numbers may not equal to 241 due to missing data.

**Table 2:** Predictors of intention to receive annual dose of COVID-19 vaccine among Chinese-Americans (*n* = 241).

	Crude PR ^a^	95% CI ^b^	Adjusted PR	95% CI
Being satisfied with previous shots of COVID-19 vaccine	1.154 ***	(1.066, 1.250)	1.104 *	(1.023, 1.191)
Perceived benefits of receiving COVID-19 vaccine	1.242 **	(1.100, 1.404)	1.123	(0.967, 1.317)
Perceived susceptibility of getting COVID-19	1.107 **	(1.035, 1.184)	1.082 *	(1.001, 1.169)
Perceived severity of getting COVID-19	1.109	(1.020, 1.206)	0.996	(0.911, 1.089)
Perceived barriers of receiving COVID-19 vaccines	0.957	(0.901, 1.016)	0.986	(0.926, 1.049)
Self-efficacy of receiving COVID-19 vaccines	1.063	(0.977, 1.156)	0.957	(0.878, 1.044)
Subjective norms	1.166	(1.055. 1.288)	1.032	(0.905, 1.176)
Cue to action (yes vs no)	1.081	(0.980, 1.192)	1.048	(0.955, 1.151)
Knowledge of COVID-19 vaccine	1.068 **	(1.023, 1.114)	1.046 *	(1.001, 1.094)
Use of the survey language (Chinese vs English)	1.042	(0.943, 1.151)	1.029	(0.926, 1.142)

^a^ Prevalence ratio; ^b^ confidence interval. * *p* < 0.05, ** *p* < 0.01, *** *p* < 0.001.

**Table 3:** Knowledge about COVID-19 vaccine among Chinese-Americans (*n* = 241).

Knowledge Items	Correct (%)
1. A COVID-19 vaccine will make me sick with COVID-19. (False)	84.3%
2. After getting a COVID-19 vaccine, I will test positive for COVID-19 on a viral test. (False)	64.3%
3. If a person already had COVID-19 and recovered, he or she still needs to be vaccinated with a COVID-19 vaccine. (True)	73.0%
4. A COVID-19 vaccine will alter my DNA. (False)	75.1%
5. It is safe for me to get a COVID-19 vaccine if I would like to have a baby one day. (True)	63.5%
6. The Pfizer-BioNTech and Moderna vaccines are mRNA vaccines. (True)	83.8%
7. The federal government is providing the vaccine free of charge to all people living in the United States, regardless of their immigration or health insurance status. (True)	92.1%

## Data Availability

As a data sharing strategy was not included in the original application for institutional review board review, study data are not publicly available.
